# The JAK1-STAT1 signaling pathway triggers inflammation responses in chronic obstructive sleep apnea rat model

**DOI:** 10.1371/journal.pone.0343053

**Published:** 2026-02-17

**Authors:** Lin Yang, Jie He

**Affiliations:** 1 Department of Pulmonary and Critical Care Medicine, Huili People’s Hospital, Liangshan Yi Autonomous Prefecture, Huili, Sichuan, China; 2 Department of Pulmonary and Critical Care Medicine, The First Affiliated Hospital of Chengdu Medical College, Chengdu, Sichuan, China; Shantou University Medical College, CHINA

## Abstract

**Background:**

Chronic obstructive sleep apnea (OSA) drives systemic inflammation; the role of the JAK1-STAT1 pathway is unclear.

**Objective:**

To elucidate JAK1-STAT1 involvement in OSA-related inflammation using a chronic intermittent hypoxia (CIH) model.

**Methods:**

Sprague-Dawley rats underwent 8-week CIH (FiO₂ cycling 5–21%, 8h/day) or normoxia (Sham). Serum cytokines (ELISA) and lung p-JAK1/p-STAT1 (Western blot/IHC) were analyzed. A CIH subset received JAK1 inhibitor filgotinib. Airway resistance was assessed via forced oscillation.

**Results:**

CIH elevated serum IL-6/TNF-α versus Sham (p < 0.05) and increased lung p-JAK1/p-STAT1. Filgotinib reduced cytokines, suppressed p-JAK1/p-STAT1, attenuated leukocyte infiltration/collagen deposition, and improved airway resistance. Lung p-STAT1 strongly correlated with serum IL-6 (r = 0.86) and TNF-α (r = 0.82) (both p < 0.001).

**Conclusion:**

JAK1-STAT1 signaling critically mediates CIH-induced inflammation. JAK1 inhibition attenuates inflammatory responses, demonstrating therapeutic potential for OSA comorbidities.

## 1. Introduction

Obstructive sleep apnea (OSA), affecting 10–17% of adults globally, imposes substantial cardiovascular and metabolic risks through chronic intermittent hypoxia (CIH), a pathological hallmark characterized by cyclic oxygen desaturation- reoxygenation events [1,2]. These hypoxic episodes trigger systemic inflammation marked by elevated interleukin-6 (IL-6) and tumor necrosis factor-alpha (TNF-α), biomarkers predictive of cardiovascular mortality in 40–60% of patients [3]. While continuous positive airway pressure (CPAP) remains first-line therapy, its limited efficacy in resolving inflammation (20–40% treatment resistance) and suboptimal adherence rates (30–60%) necessitate discovery of novel therapeutic targets addressing OSA-specific inflammatory pathways [4,5].

CIH-driven inflammation has been traditionally attributed to nuclear factor-kappa B (NF-κB) activation through redox-sensitive mechanisms akin to ischemia-reperfusion injury [6,7]. Preclinical models using FiO₂ oscillations (5–21%) confirm NF-κB’s role in endothelial dysfunction and NLRP3 inflammasome activation via NADPH oxidase-derived reactive oxygen species [8,9]. However, emerging clinical evidence reveals critical gaps in this paradigm: transcriptomic profiling of OSA patient leukocytes shows pronounced enrichment of interferon-γ (IFN-γ)/signal transducer and activator of transcription 1 (STAT1)-responsive genes, while NF-κB inhibitors only partially suppress CIH-induced cytokines in experimental models [10,11]. This discordance suggests involvement of alternative pathways, an hypothesis bolstered by recent discoveries in autoimmune research where Janus kinase 1 (JAK1)-STAT1 signaling drives macrophage inflammation independently of NF-κB [12,13]. Notably, JAK1 inhibitors like *Filgotinib*, already FDA-approved for rheumatoid arthritis, remain unexplored in OSA despite their mechanistic relevance to hypoxia-induced inflammation [14].

The JAK-STAT pathway, particularly the JAK1-STAT1 axis, emerges as a compelling candidate bridging hypoxia with inflammation [15,16]. Mechanistically, JAK1 phosphorylates STAT1 at Tyr701 upon cytokine binding (e.g., IFN-γ, IL-6), enabling nuclear translocation and activation of pro-inflammatory genes (e.g., CXCL10, SOCS1) through gamma-activated sequence elements [17–19]. Hypoxia may directly potentiate this cascade through dual mechanisms: reactive oxygen species-mediated inactivation of JAK1/STAT1-dephosphorylating protein tyrosine phosphatases, and HIF-1α-dependent JAK1 upregulation in macrophages [20,21]. Cross-disciplinary evidence supports this connection that STAT1 activation exacerbates renal inflammation in diabetes by polarizing macrophages toward pro-inflammatory M1 phenotypes, while synergizing with NF-κB to amplify IL-8 production in chronic obstructive pulmonary disease [22,23]. Despite these insights, the specific role of JAK1-STAT1 in OSA remains uncharacterized, representing a critical barrier to developing precision therapies for CPAP-resistant patients.

Addressing this gap, we employed a translational approach combining a validated CIH rat model with pharmacological inhibition using *Filgotinib*. Focusing on lung, we demonstrate for the first time that JAK1-STAT1 signaling is indispensable for CIH-induced systemic inflammation. Our findings not only establish a new mechanistic paradigm in OSA pathogenesis but also identify an immediately actionable therapeutic strategy: repurposing clinically available JAK1 inhibitors to target residual inflammation in CPAP non-responders. This work provides a roadmap for personalized management of OSA comorbidities, offering hope for high-risk patients currently underserved by standard therapies.

## 2. Materials and methods

### 2.1. Experimental animals

Male Sprague-Dawley rats (8 weeks old, 220–250 g) were purchased from Vital River Laboratories (Beijing, China; License No. SCXK 2022−0012). Rats were housed in a controlled environment (22 ± 1°C, 50 ± 5% humidity, 12-hour light/dark cycle) with ad libitum access to standard chow and water. After a 7-day acclimatization period, rats were randomly divided into three groups (n = 10 per group): [1] Normoxia control (Sham), [2] Chronic intermittent hypoxia (CIH), and [3] CIH + JAK1 inhibitor (CIH+Filgotinib). The sample size was determined a priori using G*Power 3.1 software, based on an expected large effect size (f = 0.4) in serum cytokine levels between groups, with α = 0.05 and a power (1-β) of 0.8. Baseline body weights of all groups were comparable and are provided in Supplementary S1 Table.

### 2.2. CIH protocol

The CIH model was established as previously described with modifications.^24^ Rats in the CIH and CIH+Filgotinib groups were placed in custom-designed hypoxia chambers (OxyCycler Model A84XOV, BioSpherix Ltd., USA) for 8 weeks (8 hours/day, 7 days/week). Each cycle consisted of 90 seconds of hypoxia (FiO₂ 5–6%, achieved by nitrogen infusion) followed by 90 seconds of normoxia (FiO₂ 21%), totaling 60 cycles/hour. The 90-second hypoxic/normoxic cycle parameters were selected based on a well-established CIH modeling protocol that effectively mimics the oxygen desaturation-recovery patterns observed in moderate to severe OSA [24]. Oxygen levels were continuously monitored using in-chamber sensors (ProOx 360, BioSpherix). Sham rats were maintained in identical chambers under normoxic conditions (FiO₂ 21%) for the same duration.

### 2.3.. Drug administration

*Filgotinib* (MedChemExpress, USA; Cat. No. HY-17552), a selective JAK1 inhibitor, was dissolved in 0.5% carboxymethylcellulose and administered via oral gavage at a dose of 30 mg/kg/day to the CIH+Filgotinib group 1 hour before daily CIH exposure. Sham and CIH groups received an equivalent volume of carboxymethylcellulose vehicle. This dose was selected based on prior pharmacokinetic and pharmacodynamic studies demonstrating effective JAK1 inhibition and significant anti-inflammatory efficacy in rodent models of chronic inflammation, without inducing overt toxicity [25,26].

### 2.4. Sample collection

After 8 weeks, rats were fasted overnight, anesthetized with sodium pentobarbital (50 mg/kg, intraperitoneal), and euthanized by cardiac exsanguination. Blood was collected in serum separator tubes, centrifuged (3,000 × g, 15 minutes, 4^°^C), and stored at −80°C. Lung tissues were dissected, one portion was snap-frozen in liquid nitrogen for molecular analysis, and another was fixed in 4% paraformaldehyde for histology.

### 2.5. Enzyme-linked immunosorbent assay (ELISA)

Serum levels of IL-6 and TNF-α were quantified using commercial ELISA kits (IL-6: Rat IL-6 ELISA Kit, Abcam, ab234570; TNF-α: Rat TNF-α ELISA Kit, Abcam, ab236712) following manufacturer protocols. Absorbance was measured at 450 nm (Multiskan SkyHigh, Thermo Fisher Scientific, USA). Standards and samples were run in duplicate, with intra- and inter-assay coefficients of variation <8%.

### 2.6. Western blot

Frozen tissues were homogenized in RIPA buffer (Beyotime, China) containing protease/phosphatase inhibitors (Roche, Switzerland). Protein concentrations were determined via BCA assay (Pierce, USA). Equal amounts (30 μg) were separated by SDS-PAGE, transferred to PVDF membranes (Millipore, USA), and blocked with 5% non-fat milk. Membranes were incubated overnight at 4°C with primary antibodies: anti-JAK1 (1:1,000, Cell Signaling Technology, CST#3344), anti-p-JAK1 (Tyr1034/1035; 1:1,000, CST#74129), anti-STAT1 (1:1,000, CST#14994), anti-p-STAT1 (Tyr701; 1:1,000, CST#9167), anti-STAT3 (1:1,000, CST#12640), anti-p-STAT3 (Tyr705; 1:1,000, CST#9145), and β-actin (1:5,000, CST#4970). HRP-conjugated secondary antibodies (1:5,000, CST#7074) were applied for 1 hour at room temperature. Bands were visualized using ECL reagent (Millipore) and quantified via Image Lab 6.1 (Bio-Rad, USA).

### 2.7. Immunohistochemistry

Paraffin-embedded tissues were sectioned (4 μm), deparaffinized, and subjected to antigen retrieval (citrate buffer, pH 6.0). Endogenous peroxidase activity was blocked with 3% H₂O₂. Sections were incubated with anti-p-STAT1 (1:200, CST#9167) overnight at 4°C, followed by HRP-polymer secondary antibody (ZSGB-BIO, China) and DAB substrate. Nuclei were counterstained with hematoxylin. For quantification, five random fields per section were examined at 400 × magnification. Cells were considered positively stained for p-STAT1 when distinct nuclear staining was observed at an intensity at least two-fold greater than the background. The number of positive cells per field was counted using ImageJ software (NIH, USA) by an observer blinded to the group assignments.

### 2.8. Histological analysis

All histological assessments were performed by investigators blinded to the experimental groups. H&E-stained lung sections were evaluated for leukocyte infiltration by a blinded pathologist using a semi-quantitative scoring system: 0 (none), 1 (mild), 2 (moderate), *3 (severe).* Collagen deposition was assessed by Masson’s trichrome staining. Briefly, deparaffinized and rehydrated sections were stained sequentially with: Weigert’s iron hematoxylin for 10 minutes (for nuclei), Biebrich scarlet-acid fuchsin solution for 5 minutes (for cytoplasm), followed by differentiation in phosphomolybdic-phosphotungstic acid solution for 10 minutes. Sections were then transferred directly to aniline blue solution and incubated for 10 minutes to stain collagen fibers. After brief rinsing in 1% acetic acid, sections were dehydrated, cleared, and mounted. Images were captured using a Nikon Eclipse E100 microscope, and the area of blue-stained collagen was quantified as a percentage of the total tissue area in 10 randomly selected fields per section using NIS-Elements AR 5.21 software (Nikon).

### 2.9. Airway resistance measurement

Airway resistance was assessed using the forced oscillation technique (FlexiVent FX1, SCIREQ). Rats were anesthetized with pentobarbital (50 mg/kg intraperitoneal injection), tracheostomized, and ventilated at 150 breaths/min. After recording baseline airway resistance, a methacholine challenge was performed via nebulization (25 mg/mL in PBS, Cat. #A2251, Sigma) for 3 minutes. Airway resistance was measured immediately after the challenge and at 1-minute intervals for 5 minutes. Data were analyzed using FlexiWare 8.1 software.

### 2.10. Statistical analysis

Data are expressed as mean ± SEM. Normality was assessed via Shapiro-Wilk test. Between-group differences were analyzed by one-way ANOVA with Tukey’s post hoc test (GraphPad Prism 9.0). Statistical significance was set at p < 0.05.

### 2.11. Ethical approval

All experimental procedures were approved by the Animal Ethics Committee of Chengdu Medical College (approved animal Protocol No. CMC2024MS522) according to US National Institutes of Health guidelines.

## 3. Results

### 3.1. Chronic intermittent hypoxia induces systemic inflammation

**Fig 1:**
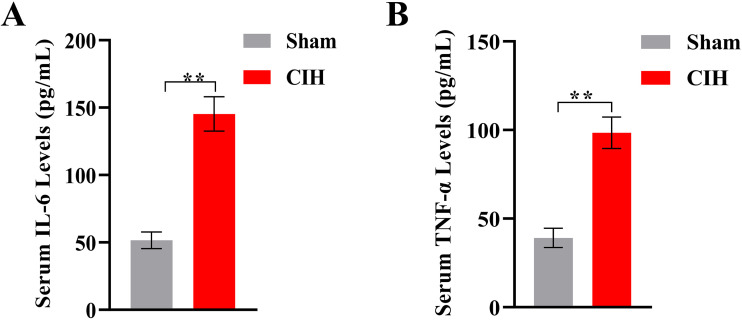
CIH-induced systemic inflammation and attenuation by JAK1 inhibition. **(A)** Serum levels of IL-6 and **(B)** TNF-α in normoxic control (Sham) and chronic intermittent hypoxia (CIH) groups. CIH exposure for 8 weeks significantly increased both cytokines compared to Sham (**p < 0.01). Data expressed as mean ± SEM; n = 8/group.

Rats exposed to 8 weeks of CIH exhibited a significant increase in systemic inflammatory cytokines compared to normoxia controls (Sham group). Serum IL-6 levels in the CIH group were elevated by 2.8-fold (CIH: 145.3 ± 12.7 pg/mL vs. Sham: 51.6 ± 6.2 pg/mL; **p < 0.01, Fig 1), while TNF-α levels increased by 2.5-fold (CIH: 98.4 ± 8.9 pg/mL vs. Sham: 39.1 ± 5.4 pg/mL; **p < 0.01) (Fig 1B). No significant differences in baseline cytokine levels were observed between groups prior to CIH exposure.

### 3.2. Activation of the JAK1-STAT1 pathway in lung tissues

**Fig 2:**
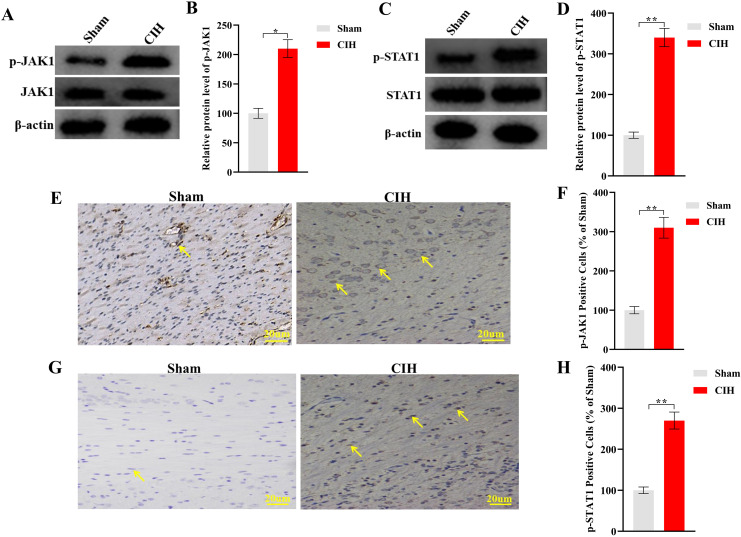
Activation of JAK1-STAT1 signaling in lung tissues after CIH. **(A-B)** Western blot analysis showing phosphorylated JAK1 protein expression (representative bands and quantitative data). **(C-D)** Western blot analysis showing phosphorylated STAT1 (Tyr701) levels (representative bands and quantitative data). **(E-F)** Immunohistochemical staining and quantification of p-JAK1-positive cells. **(G-H)** p-STAT1 immunostaining and positive cell counting. Data expressed as mean ± SEM (n = 8 rats/group). *p < 0.05, **p < 0.01 vs Sham group (unpaired t-test). Scale bars: 20 μm. Arrows indicate nuclear localization of target proteins in CIH group.

Western blot analysis revealed robust activation of the JAK1-STAT1 pathway in rats exposed to CIH. In lung tissues, JAK1 protein expression increased by 2.1-fold (*p < 0.05 vs Sham) (Fig 2A–B), and phosphorylated STAT1 (p-STAT1, Tyr701) levels were elevated by 3.4-fold (**p < 0.01 vs Sham) compared to controls (Fig 2C–D). Immunohistochemical staining further validated these findings. Lung sections from CIH rats displayed intense nuclear JAK1 (Fig 2E-F) and p-STAT1 staining (Fig 2G-H), with quantification showing a 3.1-fold (**p < 0.01 vs Sham, Fig 2F) and 2.7-fold (**p < 0.01 vs Sham, Fig 2H) increase in positive cells, respectively, compared to controls.

### 3.3. JAK1 inhibition attenuates inflammatory responses and pathway activation

**Fig 3:**
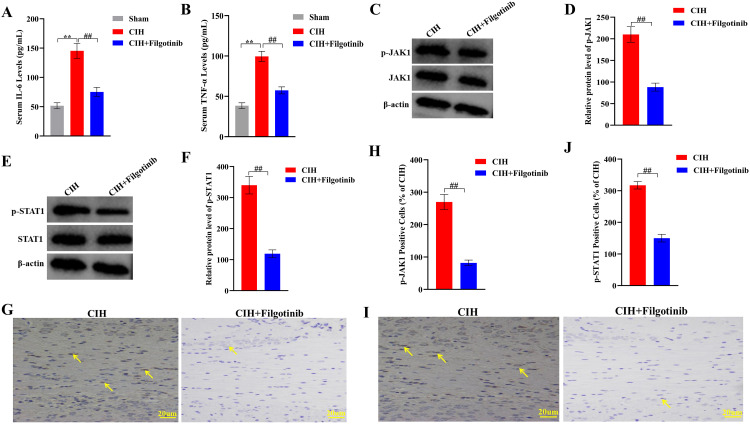
Therapeutic effects of JAK1 inhibitor *Filgotinib* on CIH-induced inflammation and JAK1-STAT1 pathway activation. **(A-B)** Serum levels of IL-6 and TNF-α. **(C-F)** Western blot analysis of p-JAK1 and p-STAT1 protein expression in lung tissues (representative bands and quantitative data). **(G-J)** Immunohistochemical staining and quantification of p-JAK1 and p-STAT1 positive cells in lung sections. Data expressed as mean ± SEM (n = 8). **p < 0.01 vs Sham group; ##p < 0.01 vs CIH group (one-way ANOVA with Tukey’s post hoc test). Scale bars: 20 μm.

Administration of the JAK1 inhibitor *Filgotinib* significantly mitigated CIH-induced inflammation. Serum IL-6 and TNF-α levels in the CIH+Filgotinib group were reduced by 48% (75.2 ± 9.1 pg/mL; ^##^p < 0.01 vs. CIH, Fig 3A) and 42% (57.3 ± 7.8 pg/mL; ^##^p < 0.01 vs. CIH, Fig 3B), respectively, approaching baseline values observed in normoxia controls (Fig 3A–B). Concurrently, *Filgotinib* treatment suppressed JAK1-STAT1 pathway activation, with the protein expression levels of p-STAT1 and JAK1 in lung tissues reduced by 65% (^##^p < 0.01, Fig 3C-D) and 58% (^##^p < 0.01, Fig 3E-F), respectively, compared to the untreated CIH group. Immunohistochemical staining for p-STAT1 and JAK1 showed reduced nuclear accumulation in the CIH+Filgotinib group, with quantification showing a 2.1-fold (^##^p < 0.01 vs CIH, Fig 3G-H) and 3.3-fold (^##^p < 0.01 vs CIH, Fig 3I-J) decrease in positive cells, respectively.

### 3.4. Histopathological improvements following JAK1 inhibition

**Fig 4:**
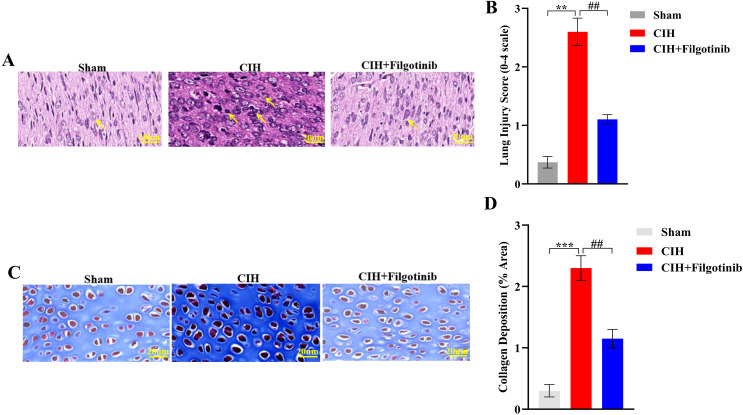
Histopathological improvements in lung tissues after JAK1 inhibition. **(A)** Representative H&E-stained lung sections (200 × magnification). Arrows indicate leukocyte infiltration. **(B)** Quantitative analysis of lung injury scores (0-4 scale). **(C)** Representative Masson’s trichrome-stained lung sections from Sham, CIH, and CIH+Filgotinib groups. Collagen fibers are stained blue. Scale bars: 20 μm. **(D)** Quantitative analysis of collagen deposition. The area of blue-stained collagen was measured as a percentage of total tissue area in 10 randomly selected fields per section using ImageJ software (NIH). Data are expressed as mean ± SEM (n = 8). ***p < 0.001 vs. Sham group; ##p < 0.01 vs. CIH group (one-way ANOVA with Tukey’s post hoc test). Data expressed as mean ± SEM (n = 8). ***p < 0.001 vs Sham group; ##p < 0.01 vs CIH group (one-way ANOVA with Tukey’s post hoc test). Scale bars: 20 μm.

Histological analysis of lung tissues corroborated the biochemical findings. CIH-exposed rats exhibited marked leukocyte infiltration in alveolar spaces (lung score: 2.6 ± 0.3 vs. Sham: 0.4 ± 0.1; **p < 0.01) (Fig 4A–B). *Filgotinib* treatment substantially reduced inflammatory cell accumulation, lowering lung scores to 1.2 ± 0.2 (##p < 0.01 vs. CIH), respectively (Fig 4A–B). Representative H&E-stained sections demonstrated preserved tissue architecture in treated rats, with minimal edema and leukocyte clustering (Fig 4A–B). Representative H&E-stained sections demonstrated preserved tissue architecture in treated rats, with minimal edema and leukocyte clustering. Similarly, Masson staining showed a 2.3-fold increase in collagen deposition in the CIH group compared to the Sham group (CIH: 2.3 ± 0.2 vs. Sham: 0.3 ± 0.1; ***p < 0.001) (Fig 4C–D). *Filgotinib* treatment substantially reduced collagen deposition, approximately 2-fold (##p < 0.01 vs. CIH, Fig 4C–D).

### 3.5. Correlation between pathway activation and inflammatory markers

**Fig 5:**
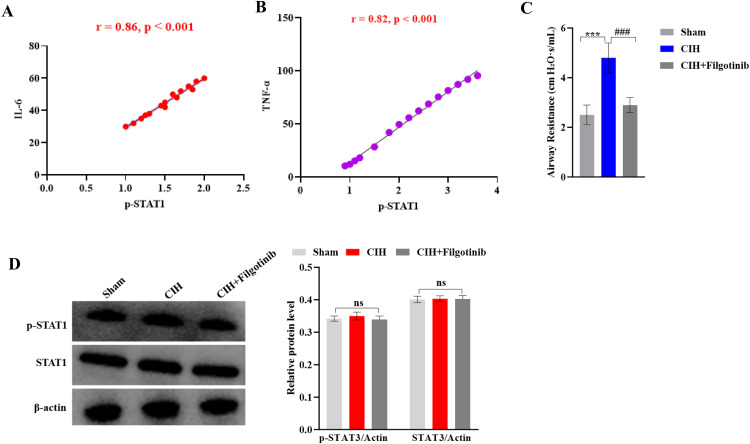
Correlation between JAK1-STAT1 activation and systemic inflammation. **(A)** Positive correlation between p-STAT1 levels in lung tissues and serum IL-6 (r = 0.86, p < 0.001, Pearson correlation, n = 15). **(B)** Positive correlation between p-STAT1 levels and serum TNF-α (r = 0.82, p < 0.001, n = 15). **(C)** Airway resistance measured by forced oscillation at baseline and after methacholine challenge. Data are presented as mean ± SEM (n = 8 per group). Baseline resistance (open bars) did not differ among groups. Post-methacholine resistance (solid bars) was significantly elevated in the CIH group compared to the Sham group (***p < 0.001). Filgotinib treatment significantly attenuated this increase (###p < 0.001 vs. CIH group; one-way ANOVA with Tukey’s post hoc test). **(D)** Analysis of STAT3 pathway activation in lung tissues. Representative Western blot bands and quantitative analysis of total STAT3 and phosphorylated STAT3 (p-STAT3, Tyr705) protein levels in the Sham and CIH groups. β-actin was used as a loading control. Data are expressed as mean ± SEM (n = 8 per group). No statistically significant difference was found between the two groups (p > 0.05, unpaired t-test).

Pearson correlation analysis revealed strong positive associations between p-STAT1 levels in lung tissues and serum IL-6 (r = 0.86, p < 0.001) and TNF-α (r = 0.82, p < 0.001) (Fig 5A–B), underscoring the systemic role of JAK1-STAT1 signaling in CIH-driven inflammation. Baseline airway resistance was comparable among all groups (Sham: 0.21 ± 0.02 cm H₂O·s/mL; CIH: 0.23 ± 0.03 cm H₂O·s/mL; CIH+Filgotinib: 0.22 ± 0.02 cm H₂O·s/mL; p > 0.05). Following methacholine challenge, functional assessment via forced oscillation demonstrated that *Filgotinib* treatment significantly attenuated the CIH-induced increase in airway resistance, normalizing it to a level comparable to the Sham group (Sham: 2.5 ± 0.4 cm H₂O·s/mL; CIH: 4.8 ± 0.6 cm H₂O·s/mL; CIH+Filgotinib: 2.9 ± 0.3 cm H₂O·s/mL; ###p < 0.001 for CIH+Filgotinib vs. CIH, Fig 5C). To confirm the specificity of the JAK1-STAT1 signaling axis in response to CIH, we examined the activation status of STAT3, another major STAT family member. Western blot analysis showed that neither the total protein level of STAT3 nor its phosphorylated form (p-STAT3, Tyr705) was significantly altered in the lung tissues of CIH-exposed rats compared to the Sham group (both p > 0.05) (Fig 5D). This result indicates that the chronic intermittent hypoxia challenge selectively activates the JAK1-STAT1, but not the JAK-STAT3, pathway.

## 4. Discussion

The present study demonstrates that CIH, a hallmark feature of OSA, triggers systemic inflammation through activation of the JAK1-STAT1 signaling axis, and pharmacological inhibition of this pathway effectively mitigates both molecular and tissue-level inflammatory damage. Our findings extend previous observations linking hypoxia to inflammatory cascades by identifying JAK1-STAT1 as a central mediator of CIH-induced pathology. While earlier studies have implicated STAT3 in sustained hypoxia responses, [25] the selective phosphorylation of STAT1 (rather than STAT3) observed here suggests a unique mechanism for intermittent hypoxia signaling. This distinction may explain the divergent cytokine profiles between CIH and chronic hypoxia models, particularly the pronounced TNF-α elevation—a feature clinically relevant to OSA-related cardiovascular complications. Mechanistically, the nuclear translocation of p-STAT1 in lung tissues aligns with its role in amplifying pro-inflammatory gene transcription.

Our results prompt a re-evaluation of the canonical inflammatory paradigm in OSA pathogenesis. While the NF-κB pathway has been extensively implicated in hypoxia-induced inflammation, largely through redox-sensitive mechanisms akin to ischemia-reperfusion injury [27–29], its predominance in the specific context of intermittent hypoxia may be incomplete. Transcriptomic profiling of leukocytes from OSA patients reveals pronounced enrichment of IFN-γ and STAT1-responsive genes, and experimental models show that NF-κB inhibitors only partially suppress CIH-induced cytokines [11,30]. Here, we observed selective activation of STAT1 (rather than STAT3) in response to CIH, suggesting a distinct signaling preference. This aligns with emerging distinctions between sustained and intermittent hypoxia; sustained hypoxia often cooperates with STAT3 and HIF-1α to drive adaptive and inflammatory responses [31,32], whereas intermittent hypoxia—through cyclic reoxygenation—may generate a reactive oxygen species signature that preferentially activates the JAK1-STAT1 axis. This mechanistic divergence could explain the unique systemic cytokine profile, particularly the robust TNF-α elevation, which is strongly linked to the cardiovascular sequelae prevalent in OSA patients.

The therapeutic efficacy of the selective JAK1 inhibitor *filgotinib* provides compelling translational validation of this pathway’s role. *Filgotinib* administration resulted in a 48% reduction in IL-6 and a 42% reduction in TNF-α, effects comparable to the anti-inflammatory benefits reported with CPAP therapy in adherent patients. Importantly, its protective effects extended beyond cytokine suppression to include attenuated leukocyte infiltration, reduced collagen deposition, and improved airway resistance. The strong positive correlations between lung p-STAT1 levels and systemic cytokines (IL-6: r = 0.86; TNF-α: r = 0.82) further suggest that JAK1-STAT1 activation is a principal driver, rather than a bystander, of CIH-induced inflammation. These findings are consistent with the established role of JAK-STAT signaling in other chronic inflammatory diseases and support the mechanistic rationale for investigating JAK inhibitors in conditions driven by intermittent hypoxia.

The clinical implications of our work are significant. Despite being the first-line therapy, CPAP fails to resolve inflammation in a substantial proportion (20−40%) of OSA patients, and adherence remains suboptimal [33,34]. Our data suggest that JAK1 inhibition could serve as a novel adjunctive or alternative strategy for CPAP non-responders or those with persistent systemic inflammation. *Filgotinib* is already FDA-approved for rheumatoid arthritis, with a well-characterized safety profile [35], which could facilitate its repurposing for OSA. Future clinical trials should consider stratifying OSA patients by inflammatory biomarkers (e.g., high-sensitivity CRP, IL-6) to identify the subgroup most likely to benefit from JAK1-targeted therapy. However, the repurposing of JAK inhibitors in an OSA population—often burdened with obesity, metabolic syndrome, and cardiovascular risk—requires careful evaluation of long-term safety, particularly regarding infection risk and metabolic parameters.

Several limitations of our study must be acknowledged. First, the 8-week CIH model, while well-validated, cannot fully recapitulate the decades-long, multifactorial progression of human OSA, which involves complex interactions with obesity, aging, and comorbidities. Second, *filgotinib* was administered prophylactically; its efficacy in reversing established inflammation or fibrosis remains to be determined. Third, our focus on lung tissue and systemic cytokines does not capture potential inflammation in other OSA-affected organ systems, such as the cardiovascular or central nervous systems. Fourth, the use of only male rats limits the generalizability of our findings. Clinical OSA exhibits sexual dimorphism, and hormones such as estrogen are known to modulate JAK-STAT signaling in other inflammatory contexts. Future studies should include both sexes to explore potential sex-dependent effects. Finally, we did not measure plasma drug concentrations, which limits pharmacokinetic- pharmacodynamic interpretations.

Future studies should prioritize three directions: 1) Delineating upstream regulators of JAK1 in CIH, particularly the potential role of pattern recognition receptors (e.g., TLR4) in sensing hypoxia-associated molecular patterns; 2) Exploring combinatorial therapies targeting both JAK1 and complementary pathways (e.g., IL-6 receptor blockade); 3) Investigating sex-specific responses, as our male-only cohort precludes generalization to female OSA populations where hormonal influences may modulate JAK-STAT activity. Clinically, our data support the rationale for repurposing JAK inhibitors in OSA patients refractory to standard therapies, though long-term safety monitoring for opportunistic infections, a known risk of JAK suppression, must accompany such trials.

## Supporting information

S1 Data This dataset will include: The individual numerical values underlying all summary statistics, graphs, and analyses presented in the manuscript (e.g., ELISA cytokine concentrations, Western blot densitometry values, histological scores, airway resistance measurements). Detailed metadata and methodology descriptions corresponding to the data files.
(XLSX)

S1 Raw Blots The original, uncropped, and unadjusted scanned images from western blots are uploaded as Supporting Information files (S2_Raw_Blots), which contain clear labeling for each lane and molecular weight marker, allowing for the verification of all cropped panels presented in the main and supplementary figures. (PDF)

S1 Table Baseline body weights. *Data presented as mean ± SEM. *p < 0.05 vs. Sham group (one-way ANOVA).
(DOCX)

## References

[1] Jordan AS, McSharry DG, Malhotra A. Adult obstructive sleep apnoea. Lancet. 2014;383(9918):736–47. doi: 10.1016/S0140-6736(13)60734-5 23910433 PMC3909558

[2] Lee JJ, Sundar KM. Evaluation and Management of Adults with Obstructive Sleep Apnea Syndrome. Lung. 2021;199(2):87–101. doi: 10.1007/s00408-021-00426-w 33713177

[3] Li X, Zhang X, Hou X, Bing X, Zhu F, Wu X, et al. Obstructive sleep apnea-increased DEC1 regulates systemic inflammation and oxidative stress that promotes development of pulmonary arterial hypertension. Apoptosis. 2023;28(3–4):432–46. doi: 10.1007/s10495-022-01797-y 36484960

[4] Benjafield AV, Pepin J-L, Cistulli PA, Wimms A, Lavergne F, Sert Kuniyoshi FH, et al. Positive airway pressure therapy and all-cause and cardiovascular mortality in people with obstructive sleep apnoea: a systematic review and meta-analysis of randomised controlled trials and confounder-adjusted, non-randomised controlled studies. Lancet Respir Med. 2025;13(5):403–13. doi: 10.1016/S2213-2600(25)00002-5 40118084 PMC12045716

[5] DeVettori G, Troxel WM, Duff K, Baron KG. Positive airway pressure adherence among patients with obstructive sleep apnea and cognitive impairment: A narrative review. Sleep Med. 2023;111:28–35. doi: 10.1016/j.sleep.2023.08.029 37716335 PMC10613340

[6] Que Y, Meng H, Ding Y, Fan J, Du Y, Xu G. Investigation of the shared gene signatures and molecular mechanisms between obstructive sleep apnea syndrome and asthma. Gene. 2024;896:148029. doi: 10.1016/j.gene.2023.148029 38007161

[7] Dandan Z, Shen C, Liu X, Liu T, Ou Y, Ouyang R. IL-33/ST2 mediating systemic inflammation and neuroinflammation through NF-kB participated in the neurocognitive impairment in obstructive sleep apnea. Int Immunopharmacol. 2023;115:109604. doi: 10.1016/j.intimp.2022.109604 36580760

[8] Oba K, Yamashita H, Waragai A, Kawano T. NF-kappaB in the lungs of premature rabbits during mechanical ventilation--comparison between conventional mechanical ventilation (CMV) and high-frequency oscillation (HFO). Pediatr Pulmonol. 2007;42(5):446–51. doi: 10.1002/ppul.20580 17394254

[9] Tung Y-T, Wei C-H, Yen C-C, Lee P-Y, Ware LB, Huang H-E, et al. Aspirin Attenuates Hyperoxia-Induced Acute Respiratory Distress Syndrome (ARDS) by Suppressing Pulmonary Inflammation via the NF-κB Signaling Pathway. Front Pharmacol. 2021;12:793107. doi: 10.3389/fphar.2021.793107 35111059 PMC8802116

[10] Cortese R, Adams TS, Cataldo KH, Hummel J, Kaminski N, Kheirandish-Gozal L, et al. Single-cell RNA-seq uncovers cellular heterogeneity and provides a signature for paediatric sleep apnoea. Eur Respir J. 2023;61(2):2201465. doi: 10.1183/13993003.01465-2022 36356973

[11] Ni W, Niu Y, Cao S, Fan C, Fan J, Zhu L, et al. Intermittent hypoxia exacerbates anxiety in high-fat diet-induced diabetic mice by inhibiting TREM2-regulated IFNAR1 signaling. J Neuroinflammation. 2024;21(1):166. doi: 10.1186/s12974-024-03160-1 38956653 PMC11218348

[12] Zhang J, Xu Y, Wei C, Yin Z, Pan W, Zhao M, et al. Macrophage neogenin deficiency exacerbates myocardial remodeling and inflammation after acute myocardial infarction through JAK1-STAT1 signaling. Cell Mol Life Sci. 2023;80(11):324. doi: 10.1007/s00018-023-04974-7 37824022 PMC11072237

[13] Ryu S, Sidorov S, Ravussin E, Artyomov M, Iwasaki A, Wang A, et al. The matricellular protein SPARC induces inflammatory interferon-response in macrophages during aging. Immunity. 2022;55(9):1609–26.e7. doi: 10.1016/j.immuni.2022.07.007 35963236 PMC9474643

[14] Kerschbaumer A, Sepriano A, Smolen JS, van der Heijde D, Dougados M, van Vollenhoven R, et al. Efficacy of pharmacological treatment in rheumatoid arthritis: a systematic literature research informing the 2019 update of the EULAR recommendations for management of rheumatoid arthritis. Ann Rheum Dis. 2020;79(6):744–59. doi: 10.1136/annrheumdis-2019-216656 32033937 PMC7286044

[15] Xu J, Wakai M, Xiong K, Yang Y, Prabakaran A, Wu S, et al. The pro-inflammatory cytokine IL6 suppresses mitochondrial function via the gp130-JAK1/STAT1/3-HIF1α/ERRα axis. Cell Rep. 2025;44(3):115403. doi: 10.1016/j.celrep.2025.115403 40056415

[16] Wang F, Xia J-J, Shen L-J, Jiang T-T, Li W-L, You D-L, et al. Curcumin attenuates intracerebral hemorrhage-induced neuronal apoptosis and neuroinflammation by suppressing JAK1/STAT1 pathway. Biochem Cell Biol. 2022;100(3):236–45. doi: 10.1139/bcb-2021-0423 35381181

[17] Palmer MA, Kirchhoff R, Buerger C, Benatzy Y, Schebb NH, Brüne B. RNAi-based ALOX15B silencing augments keratinocyte inflammation in vitro via EGFR/STAT1/JAK1 signalling. Cell Death Dis. 2025;16(1):39. doi: 10.1038/s41419-025-07357-x 39843435 PMC11754432

[18] Xue C, Yao Q, Gu X, Shi Q, Yuan X, Chu Q, et al. Evolving cognition of the JAK-STAT signaling pathway: autoimmune disorders and cancer. Signal Transduct Target Ther. 2023;8(1):204. doi: 10.1038/s41392-023-01468-7 37208335 PMC10196327

[19] Montero P, Milara J, Roger I, Cortijo J. Role of JAK/STAT in Interstitial Lung Diseases; Molecular and Cellular Mechanisms. Int J Mol Sci. 2021;22(12). 10.3390/ijms22126211 PMC8226626 34207510

[20] Wang Y, Wang D, Zhang L, Ye F, Li M, Wen K. Role of JAK-STAT pathway in reducing cardiomyocytes hypoxia/reoxygenation injury induced by S1P postconditioning. Eur J Pharmacol. 2016;784:129–36. doi: 10.1016/j.ejphar.2016.05.024 27215146

[21] Fearon U, Canavan M, Biniecka M, Veale DJ. Hypoxia, mitochondrial dysfunction and synovial invasiveness in rheumatoid arthritis. Nat Rev Rheumatol. 2016;12(7):385–97. doi: 10.1038/nrrheum.2016.69 27225300

[22] Li C, Ma Q-Y, Liu X-Q, Li H, Yu M-J, Xie S-S, et al. Genetic and pharmacological inhibition of GRPR protects against acute kidney injury via attenuating renal inflammation and necroptosis. Mol Ther. 2023;31(9):2734–54. doi: 10.1016/j.ymthe.2023.06.016 37415332 PMC10492025

[23] Clarke DL, Clifford RL, Jindarat S, Proud D, Pang L, Belvisi M, et al. TNFα and IFNγ synergistically enhance transcriptional activation of CXCL10 in human airway smooth muscle cells via STAT-1, NF-κB, and the transcriptional coactivator CREB-binding protein. J Biol Chem. 2010;285(38):29101–10. doi: 10.1074/jbc.M109.0999952 20833730 PMC2937941

[24] Li L, Ren F, Qi C, Xu L, Fang Y, Liang M, et al. Intermittent hypoxia promotes melanoma lung metastasis via oxidative stress and inflammation responses in a mouse model of obstructive sleep apnea. Respir Res. 2018;19(1):28. doi: 10.1186/s12931-018-0727-x 29433520 PMC5809953

[25] Inoue S, Ikai M, Nambu R, Moriya K, Kojima R, Tagami Y, et al. JAK inhibitor ameliorates inflammatory bowel disease in a patient with IKZF1 haploinsufficiency. Clin Immunol. 2025;274:110470. doi: 10.1016/j.clim.2025.110470 40037506

[26] O’Brien A, Hanlon MM, Marzaioli V, Wade SC, Flynn K, Fearon U, et al. Targeting JAK-STAT Signalling Alters PsA Synovial Fibroblast Pro-Inflammatory and Metabolic Function. Front Immunol. 2021;12:672461. 34248953 10.3389/fimmu.2021.672461 PMC8264423

[27] Li M, Ying M, Gu S, Zhou Z, Zhao R. Matrine alleviates hypoxia-induced inflammation and pulmonary vascular remodelling via RPS5/NF-κB signalling pathway. J Biochem Mol Toxicol. 2024;38(1):e23583. doi: 10.1002/jbt.23583 37986032

[28] Pham K, Parikh K, Heinrich EC. Hypoxia and Inflammation: Insights From High-Altitude Physiology. Front Physiol. 2021;12:676782. doi: 10.3389/fphys.2021.676782 34122145 PMC8188852

[29] Arnaud C, Billoir E, de Melo Junior AF, Pereira SA, O’Halloran KD, Monteiro EC. Chronic intermittent hypoxia-induced cardiovascular and renal dysfunction: from adaptation to maladaptation. J Physiol. 2023;601(24):5553–77. doi: 10.1113/JP284166 37882783

[30] de Lima FFF, Mazzotti DR, Tufik S, Bittencourt L. The role inflammatory response genes in obstructive sleep apnea syndrome: a review. Sleep Breath. 2016;20(1):331–8. doi: 10.1007/s11325-015-1226-7 26201496

[31] Huynh L, Kusnadi A, Park SH, Murata K, Park-Min K-H, Ivashkiv LB. Opposing regulation of the late phase TNF response by mTORC1-IL-10 signaling and hypoxia in human macrophages. Sci Rep. 2016;6:31959. doi: 10.1038/srep31959 27558590 PMC4997257

[32] Cetin-Atalay R, Meliton AY, Tian Y, Sun KA, Woods PS, Shin KWD, et al. Sustained hypoxia but not intermittent hypoxia induces HIF-1α transcriptional response in human aortic endothelial cells. Mol Omics. 2025;21(1):19–31. doi: 10.1039/d4mo00142g 39513671 PMC11563308

[33] Askland K, Wright L, Wozniak DR, Emmanuel T, Caston J, Smith I. Educational, supportive and behavioural interventions to improve usage of continuous positive airway pressure machines in adults with obstructive sleep apnoea. Cochrane Database Syst Rev. 2020;4(4):CD007736. doi: 10.1002/14651858.CD007736.pub3 32255210 PMC7137251

[34] Tselepi C, Tsirves G, Exarchos K, Chronis C, Kyriakopoulos C, Tatsis K, et al. Educational video demonstrating collapsibility of the upper airway during sleep improves initial acceptance of CPAP in patients with severe obstructive sleep apnea: a retrospective study. J Clin Sleep Med. 2024;20(9):1423–33. doi: 10.5664/jcsm.11166 38648113 PMC11367730

[35] Combe B, Kivitz A, Tanaka Y, van der Heijde D, Simon JA, Baraf HSB, et al. Filgotinib versus placebo or adalimumab in patients with rheumatoid arthritis and inadequate response to methotrexate: a phase III randomised clinical trial. Ann Rheum Dis. 2021;80(7):848–58. doi: 10.1136/annrheumdis-2020-219214 33504485 PMC8237199

