# Supplementary material for: The JAK1-STAT1 signaling pathway triggers inflammation responses in chronic obstructive sleep apnea rat model
Source: PLoS One. 2026 Feb 17;21(2):e0343053. doi: 10.1371/journal.pone.0343053 (PMC12912577; doi:10.1371/journal.pone.0343053)
Supplement: S1 Raw Blots — (PDF) [file pone.0343053.s002.pdf]

Fig 2A

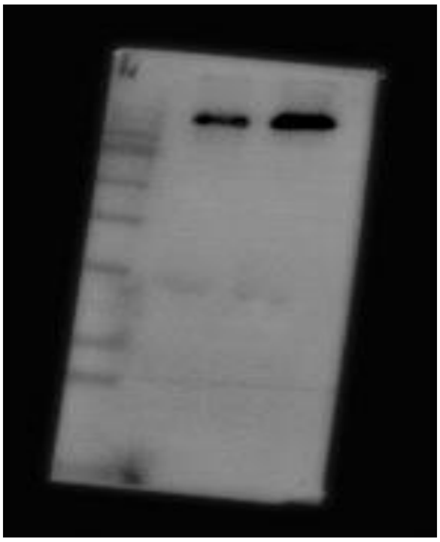

p-JAK1

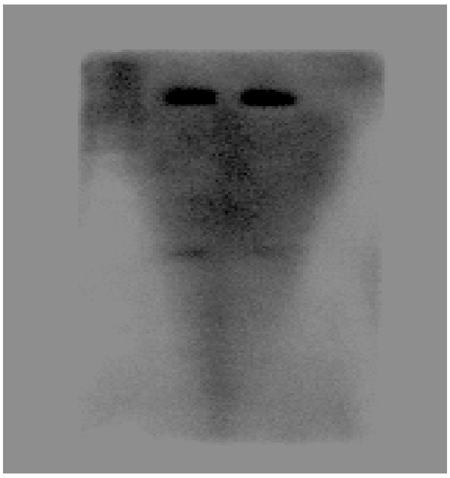

JAK1

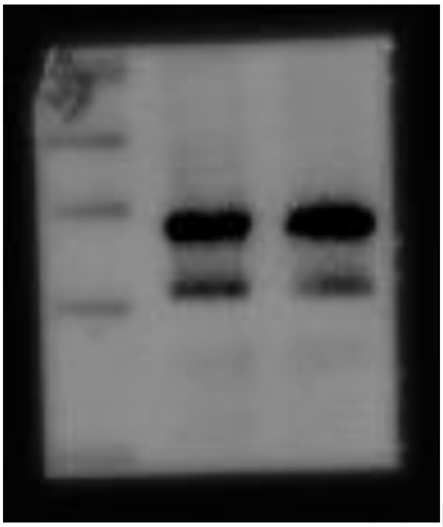

β-actin

Fig 2C

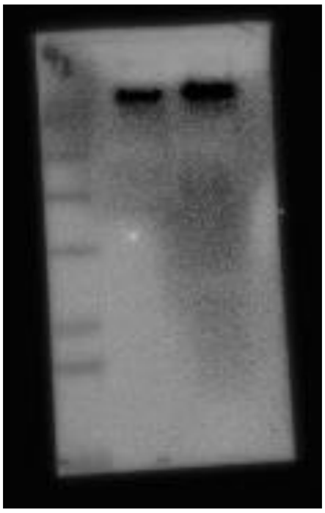

p-STAT1

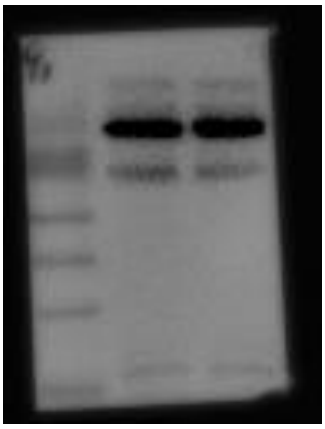

STAT1

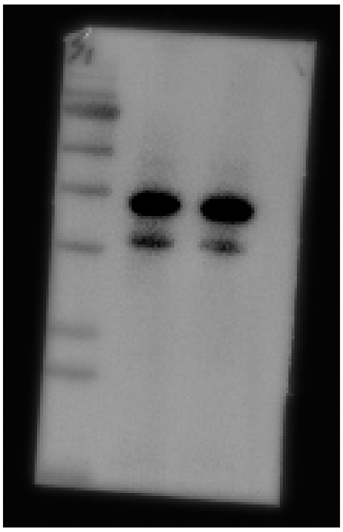

β-actin

Fig 3C

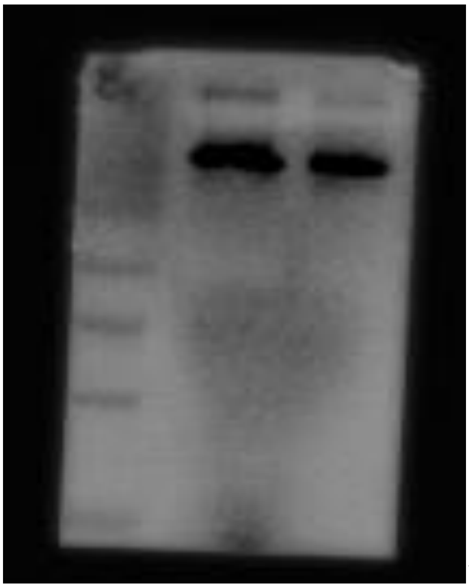

p-JAK1

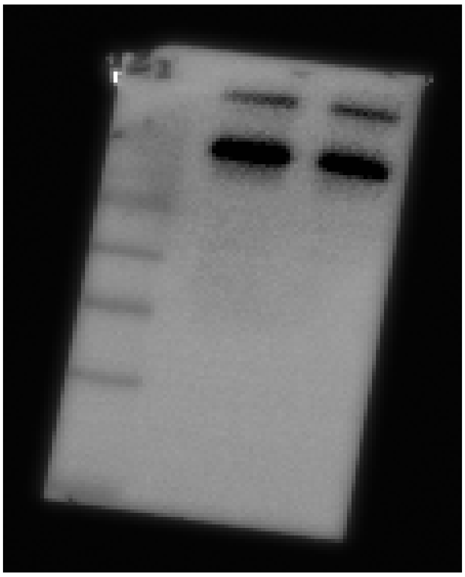

JAK1

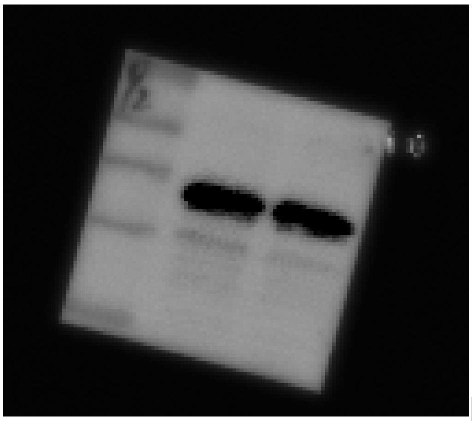

β-actin

Fig 3E

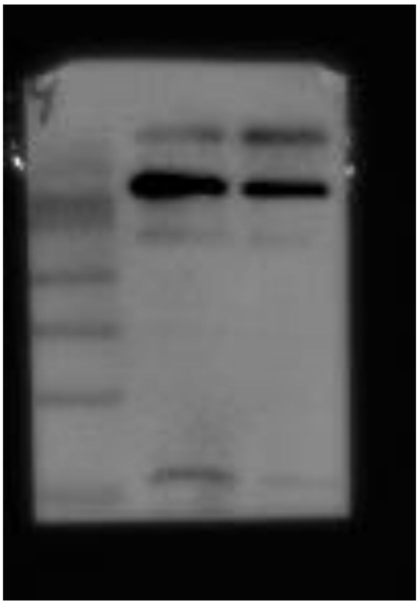

p-STAT1

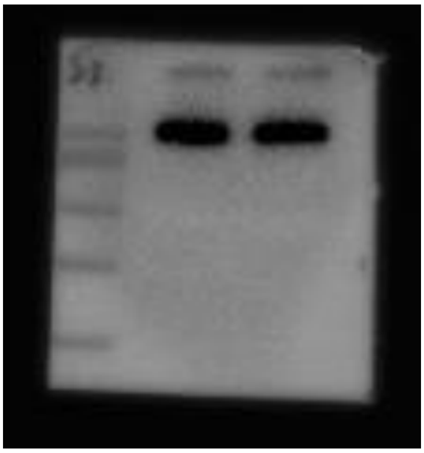

STAT1

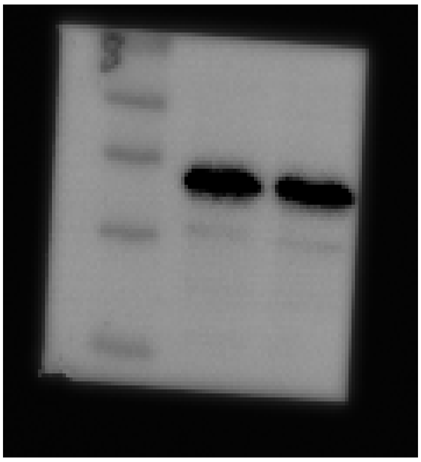

β-actin

Fig 5D

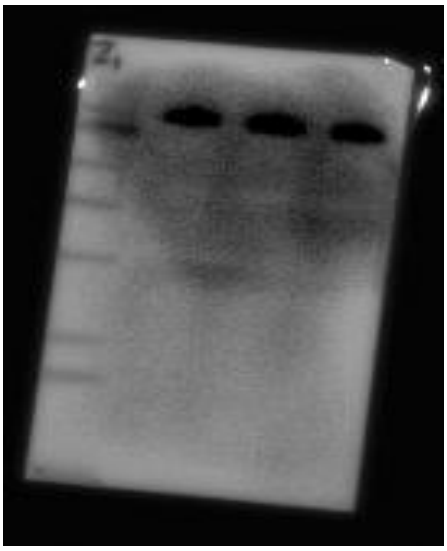

p-STAT1

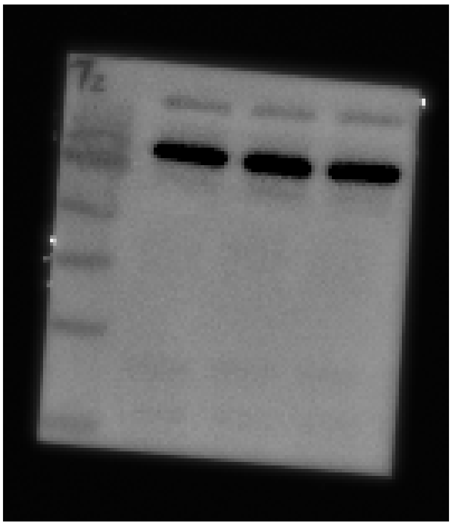

STAT1

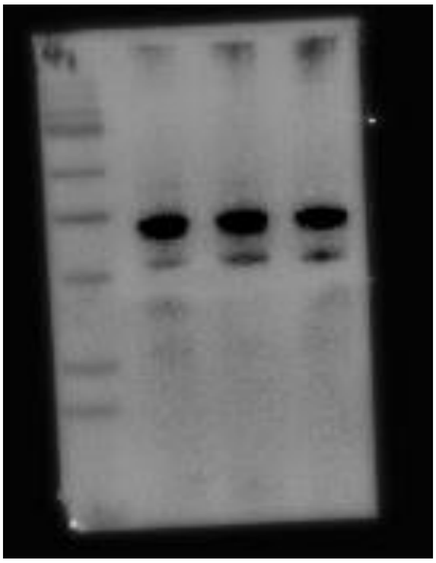

β-actin
