# Supplementary material for: The JAK1-STAT1 signaling pathway triggers inflammation responses in chronic obstructive sleep apnea rat model
Source: PLoS One. 2026 Feb 17;21(2):e0343053. doi: 10.1371/journal.pone.0343053 (PMC12912577; doi:10.1371/journal.pone.0343053)
Supplement: S1 Table — *Data presented as mean ± SEM. *p < 0.05 vs. Sham group (one-way ANOVA). (DOCX) [file pone.0343053.s003.docx]

**Supplementary Table S1**

| **Group** | **n** | **Initial Body Weight (g)** | **Final Body Weight (g)** |
| --- | --- | --- | --- |
| Sham (Normoxia) | 10 | 228.5 ± 5.2 | 512.3 ± 18.7 |
| CIH | 10 | 230.1 ± 4.8 | 485.6 ± 20.1* |
| CIH + Filgotinib | 10 | 229.3 ± 5.5 | 498.4 ± 19.5 |

*Data presented as mean ± SEM. *p < 0.05 vs. Sham group (one-way ANOVA).
